# Combination Therapy With MET Tyrosine Kinase Inhibitor and EGFR Tyrosine Kinase Inhibitor in Patients With MET-Overexpressed EGFR-Mutant Lung Adenocarcinoma

**DOI:** 10.1016/j.jtocrr.2025.100832

**Published:** 2025-04-09

**Authors:** Jia-Jun Wu, Zhe-Rong Zheng, Tse-Hsien Lo, Cheng-Hsiang Chu, Kun-Chieh Chen, Gee-Chen Chang

**Affiliations:** a Institute of Medicine, Chung Shan Medical University, Taichung, Taiwan; b School of Medicine, Chung Shan Medical University, Taichung, Taiwan; c Division of Pulmonary Medicine, Department of Internal Medicine, Chung Shan Medical University Hospital, Taichung, Taiwan; d Institute of Biomedical Sciences, National Chung Hsing University, Taichung, Taiwan

**Keywords:** EGFR mutation, EGFR tyrosine kinase inhibitor, Lung adenocarcinoma (LUAD), MET overexpression, MET tyrosine kinase inhibitor, Non–small cell lung cancer (NSCLC)

## Abstract

**Introduction:**

Dysregulated *MET* signaling, such as MET overexpression or *MET* amplification (*MET*amp), is a important mechanism of resistance to EGFR tyrosine kinase inhibitors (TKIs) in patients with *EGFR*-mutant lung adenocarcinoma (LUAD). Combination therapy with EGFR TKIs and MET TKIs has revealed efficacy in these patients. This study aimed to analyze the real-world experience of TKI combination in patients with *EGFR*-mutant MET-overexpressed LUAD.

**Methods:**

This retrospective cohort study included patients with advanced *EGFR*-mutant LUAD who progressed after EGFR TKIs and were treated with combination therapy of EGFR TKIs and MET TKIs (capmatinib or tepotinib). Immunohistochemistry was used to detect MET overexpression.

**Results:**

This study included 27 patients, with a median age of 69 years; 40.7% of the patients were male individuals, and 88.9% never smoked. Overall, the treatment response of the TKI combination reported 29.6% (eight of 27) partial response, 55.6% (15 of 27) stable disease, a median progression-free survival of 7.3 months, and an overall survival of 26.9 months. The adverse events were mostly grade 1 to 2, with only one patient experiencing a grade 3 or greater event, which was peripheral edema. The most common adverse events were hypoalbuminemia (44.4%), increased creatinine (44.4%), and peripheral edema (44.4%). Eight patients underwent next-generation sequencing analysis, and two (25.0%) of them had *MET*amp. Three patients (37.5%) had *TP53* mutations, which were the most common concurrent alterations. Those with positive *MET*amp had significantly longer median progression-free survival than those without (25.3 versus 5.8 mo; *p* = 0.034).

**Conclusions:**

The TKI combination reported clinical activities in patients with advanced *EGFR*-mutant LUAD resistant to EGFR TKIs and mild toxicity in those with MET overexpression.

## Introduction

Lung cancer is the leading cause of cancer-related mortality worldwide.^1^ *EGFR* mutations are one of the most common driver gene alterations (15%–55%) in patients with NSCLC.^2^^,^^3^ Although EGFR tyrosine kinase inhibitors (TKIs) have been approved as first-line treatment in patients with metastatic *EGFR*-mutant NSCLC, most patients eventually develop drug resistance.^4^^,^^5^

The mechanism of resistance to EGFR TKI can be categorized into on-target and off-target mechanisms.^4^ For those who received first- or second-generation EGFR TKIs as first-line treatment, on-target EGFR-T790M mutations (40%–55%) were the most common resistance mechanism, whereas off-target mechanisms accounted for most acquired resistance (70%–80%) after progression to osimertinib.^4^ MET signaling may become abnormal or dysregulated through various mechanisms, including the overexpression of the MET protein or genetic alterations in the MET gene, such as mutations, amplifications, or rearrangements.^6^^,^^7^ In patients with *EGFR*-mutant NSCLC, the alterations in MET signaling, including *MET* gene amplification (*MET*amp) and MET overexpression, are significant mechanisms of acquired resistance to EGFR TKIs, through the activation of *EGFR*-independent phosphorylation of ErbB3 and downstream activation of the PI3K/AKT pathway.^8^^,^^9^ *MET*amp are composed of 7% to 18% of EGFR acquired resistance mechanisms, whereas the incidence of MET overexpression has not been clearly reported, which may be due to the difficulty in defining normal or overexpression.^10^ In addition, the detection of MET overexpression was not well associated with *MET*-dependent activations.^11^^,^^12^

For patients with *MET*-altered NSCLC, several MET TKIs have shown therapeutic activities against these tumors.^13^, ^14^, ^15^ Recently, combination therapy with EGFR TKI and MET TKI has revealed efficacy in patients with MET-overexpressed or amplified, *EGFR*-mutant NSCLC with disease progression on prior EGFR TKI.^16^, ^17^, ^18^ Nevertheless, treatment response and diagnostic criteria of MET alterations varied between different studies. In addition, the real-world experience of TKI combination therapy in patients with MET overexpression remained scarce. Therefore, in this study, we aimed to analyze the treatment efficacies and toxicities of combination therapy in patients with advanced *EGFR*-mutant lung adenocarcinoma (LUAD) with MET overexpression after progression from EGFR TKI treatment in the real-world setting.

## Materials and Methods

### Patients

This study was a retrospective, single-center, observational study at Chung Shan Medical University Hospital in Taiwan. The study was conducted ethically in accordance with the World Medical Association Declaration of Helsinki and was approved by the Institutional Review Board of Chung Shan Medical University Hospital, Taiwan, and written informed consent documents for genetic testing, biomarkers including immunohistochemistry (IHC) tests, and clinical data records were obtained from all patients (Institutional Review Board Number CS1-20105).

We enrolled *EGFR*-mutant LUAD patients between June 2020 and October 2023. To be eligible for the study, patients had to fulfill the following inclusion criteria: a diagnosis of histologically and cytologically confirmed LUAD, recurrent or inoperable advanced stage IIIB or C to stage IV lung cancer according to the eighth edition of the American Joint Committee for Cancer TNM staging system, *EGFR* mutation, and treatment with first-line EGFR TKIs, including gefitinib, erlotinib, afatinib, and osimertinib. Patients who had progressive disease to EGFR TKIs, had positive MET overexpression as acquired resistance to EGFR TKI, were included in the analysis of the clinical efficacy of combination treatment with EGFR TKIs and MET TKIs (capmatinib or tepotinib). Patients were excluded if they had a co-mutation with *EGFR* exon 20 insertion or had been diagnosed with another malignancy under treatment. Patients simultaneously receiving combination therapy including chemotherapy, antiangiogenetic agents, or any anticancer drugs in addition to MET TKIs and EGFR TKIs were excluded from the analysis. Patients receiving combination therapy of EGFR TKI with MET TKI after progression on chemotherapy were included in the analysis (i.e., patients who received chemotherapy as second-line treatment after progression on EGFR TKI and then received TKI combination therapy as later-line treatment after progression on chemotherapy). The treatment response was evaluated by the Response Evaluation Criteria in Solid Tumors (RECIST) version 1.1.

Each patient’s demographic and clinical data, including age, sex, smoking status, Eastern Cooperative Oncology Group performance status, baseline *EGFR* mutation status, results of tumor molecular study, type of EGFR TKI treatment, response to combination therapy of EGFR TKIs with MET TKIs, progression-free survival (PFS) and overall survival (OS), were collected for analysis. The response of a tumor to the combination treatment was evaluated according to RECIST version 1.1. The adverse events (AEs) of combination treatment were reported on the basis of the Common Terminology Criteria for Adverse Events version 5.0.

### EGFR Mutation, MET IHC, and Tumor Molecular Study for Tumor Tissue Rebiopsy

In this study, all patients underwent rebiopsy to determine the mechanism of acquired resistance. Tissue was obtained from lung tumors through various approaches, including surgery, bronchoscopic biopsy, ultrasound-guided percutaneous tumor biopsy, or computed tomography–guided biopsy. In addition, surgical removal of metastatic lymph nodes for examination was permitted.

The *EGFR* mutation tests were from certified methods, including polymerase chain reaction (PCR) or next-generation sequencing (NGS) tests. For the PCR assay, genomic DNA was extracted from formalin-fixed, paraffin-embedded sections of tumor tissues using xylene deparaffinization and an optimized Cobas DNA Sample Preparation Kit. DNA sequences at 42 targets within exon 18, 19, 20 and 21 of the EGFR gene were analyzed using the Cobas z 480 Analyzer (real-time PCR) and Cobas EGFR Mutation Test version 2 Kit (Roche Molecular Diagnostics, Pleasanton, CA).

In this study, either tissue or liquid NGS assays were permitted. For tissue NGS, formalin-fixed, paraffin-embedded samples were analyzed using the FoundationOne CDx (Foundation Medicine, Cambridge, MA), Oncomine Comprehensive Assay version 3 (Thermo Fisher Scientific, Waltham, MA), or ACTOnco+ comprehensive cancer gene panel (ACT Genomics, Taiwan). For liquid NGS, plasma samples were examined using the Guardant360 CDx (Guardant Health, Palo Alto, CA).

MET IHC was performed in rebiopsy tissues after EGFR TKI treatment failure using EP1454Y antibody and the benchmark XT (Ventana) platform, and the examples are revealed in Supplementary Figure 1. MET expression was defined as an IHC 0 to 3 or higher scale into four degrees: 3 or higher (≥50% tumor cells exhibit strong staining intensity), 2 or higher (≥50% tumor cells reveal moderate or higher staining intensity, but less than 50% exhibit strong intensity), 1 or higher (≥50% tumor cells display weak or higher staining intensity, but less than 50% reveal moderate or higher intensity), and 0 (either no staining or less than 50% of tumor cells with any staining intensity). Per the MetMab criteria, the cutoff scale for MET overexpression should be at least 2 or higher or 3 or higher.^19^ *MET*amp were detected by NGS tests of tissue or liquid biopsy and were defined as positive according to the criteria of each panel.

### Statistical Methods

Data are presented as frequencies (percentages) for categorical variables and medians (interquartile range) for continuous variables. To determine the differences between groups, the chi-square or Fisher’s exact test was applied for categorical variables and the Mann-Whitney *U* test for continuous variables. PFS was measured as the time from the first dose of the combination treatment of EGFR TKI and MET TKI to progression or death. OS was measured as the time from the first dose of the combination treatment with EGFR TKI and MET TKI to death from any cause. Patients were censored if they were alive at the time of analysis during the last follow-up. The Kaplan-Meier method was used to estimate the survival time. Differences in survival time were analyzed using the log-rank test. Univariate and multivariate analyses of survival outcomes were performed with a Cox proportional hazards model. Two-tailed tests with *p* values less than 0.05 were considered statistically significant. All analyses were performed using the IBM SPSS Statistics package, version 29 (IBM Corporation, Armonk, NY).

## Results

### Patient Characteristics

**Figure 1:**
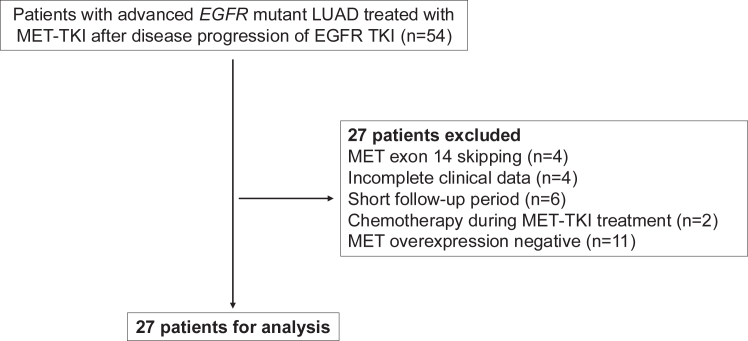
Flow chart of patient inclusion. LUAD, lung adenocarcinoma; TKI, tyrosine kinase inhibitor.

Overall, 54 patients with advanced *EGFR*-mutant LUAD treated with MET TKI after disease progression of EGFR TKI were screened for eligibility. Twenty-seven patients were analyzed after excluding 27 patients who did not meet the inclusion criteria (Fig. 1). The demographics of the patients are shown in Table 1. The median age of the patients was 69 years. Of them, 11 (40.7%) were male individuals, 24 (88.9%) never smoked, and three (11.1%) had brain metastasis. Eleven patients (40.7%) had exon 19 deletion, 14 (51.9%) had L858R initially, and two (7.4%) had an uncommon mutation, whereas 11 (40.7%) had T790M as acquired *EGFR* mutation. Fourteen patients (51.9%) underwent chemotherapy before MET inhibitor treatment. Twelve patients (44.4%) were tested with MET IHC 2 or higher, and 15 (55.6%) with 3 or higher. Before combination therapy, eight of the 27 patients (29.6%) received first- or second-generation EGFR TKI as frontline treatment, and three out of the eight patients had EGFR TKI switched to osimertinib when they received combination therapy. Regarding the combination treatment, 22 patients (81.5%) received capmatinib and five (18.5%) received tepotinib as the MET inhibitor; six patients (22.2%) received first- or second-generation EGFR TKI, and 21 (77.8%) received osimertinib as EGFR TKI.

**Table 1:** Patients’ Demographics

Variables	All (N = 27)
Age (y), median (IQR)	69 (60–71)
Sex	
Male	11 (40.7)
Female	16 (59.3)
Smoking	
Never smoked	24 (88.9)
Smoked	3 (11.1)
ECOG PS	
0–1	24 (88.9)
≥2	3 (11.1)
Stage	
IVA	11 (40.7)
IVB	16 (59.3)
Brain metastasis	3 (11.1)
*EGFR* mutation	
Del 19	11 (40.7)
L858R	14 (51.9)
Uncommon mutation^a^	2 (7.4)
T790M^b^	11 (40.7)
*MET* overexpression	
2+	12 (44.4)
3+	15 (55.6)
Consolidation therapy to primary tumor^c^	12 (44.4)
Chemotherapy before MET TKI	14 (51.9)
MET inhibitor	
Capmatinib	22 (81.5)
Tepotinib	5 (18.5)
EGFR inhibitor^d^	
First or second generation	6 (22.2)
Third generation	21 (77.8)

*Note:* Categorical data are presented as n (%).

Del 19, exon 19 deletion; ECOG PS, Eastern Cooperative Oncology Group performance status; IQR, interquartile range; TKI, tyrosine kinase inhibitor.

a One had G719R plus S768I plus V769L; one had G719X.

b Aquired mutation.

c Surgical resection or radiotherapy to primary tumor.

d First or second generation refers to gefitinib, erlotinib, or afatinib; third generation refers osimertinib.

### Treatment Response to the TKI Combination and Safety Profiles

**Figure 2:**
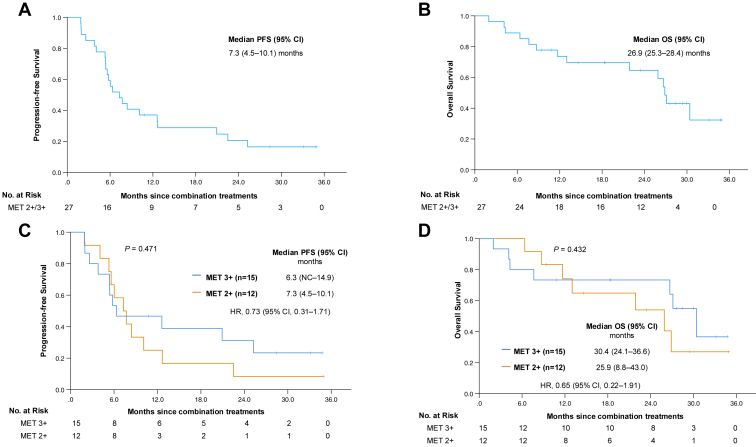
PFS *(A,C)* and OS *(B,D)* of TKI combination (EGFR TKI and MET TKI) comparing between those with MET IHC 2 or higher and 3 or higher. CI, confidence interval; IHC, immunohistochemistry; NC, could not be calculated; OS, overall survival; PFS, progression-free survival; TKI, tyrosine kinase inhibitor.

In general, the median duration of follow-up was 21.9 months (interquartile range: 9.4–28.4 mo). The treatment response is revealed in Table 2, with an objective response rate (ORR) of 29.6% and a disease control rate (DCR) of 85.2%. The median PFS (mPFS) was 7.3 months (95% confidence interval: 4.5–10.1), whereas the median OS (mOS) was 26.9 months (95% confidence interval: 25.3–28.4) (Fig. 2*A*–*D*). Patients with MET IHC 2 or higher and 3 or higher reported similar ORR (25.0% versus 33.3%; *p* = 0.696), DCR (91.7% versus 80.0%; *p* = 0.605), mPFS (7.3 versus 6.3 mo; *p* = 0.471), and mOS (25.9 versus 30.4 mo; *p* = 0.432). Univariate and multivariate analyses found no independent factors associated with PFS or OS in this study (Supplementary Table 1).

**Table 2:** Patient’s Outcomes of the Treatment of MET TKI

Outcomes	All	MET 3+	MET 2+	*p* Value^a^
	N = 27	n = 15	n = 12	
Survival time, median (95% CI)				
PFS, mo	7.3 (4.5–10.1)	6.3 (NC–14.9)	7.3 (4.5–10.1)	0.471
OS, mo	26.9 (25.3–28.4)	30.4 (24.1–36.6)	25.9 (8.8–43.0)	0.432
Treatment response, n (%)				
PR	8 (29.6)	5 (33.3)	3 (25.0)	0.536
SD	15 (55.6)	7 (46.7)	8 (66.7)	
PD	4 (14.8)	3 (20.0)	1 (8.3)	
ORR	29.6%	33.3%	25.0%	0.696
DCR	85.2%	80.0%	91.7%	0.605

CI, confidence interval; DCR, disease control rate; NC, could not be calculated; ORR, objective response rate; OS, overall survival; PD, progressive disease; PFS, progression-free survival; PR, partial response; SD, stable disease; TKI, tyrosine kinase inhibitor.

a *p* value: By log rank test for survival time; by Fisher’s exact test for treatment response.

The presence of AEs from the combination treatment is listed in Table 3. The most common side effects were hypoalbuminemia (44.4%), peripheral edema (44.4%), increased creatinine (44.4%), and skin rash (29.6%). Only one patient (2.8%) experienced a grade 3 or higher AE, which was peripheral edema. Among the 11 patients who had peripheral edema, 10 had diuretic therapy. Five patients received a half dose of MET TKI initially (four had a 200-mg twice daily dose of capmatinib, and one had a 250-mg once daily dose of tepotinib), and none had a dose adjustment during the treatment course. Overall, there were no events causing discontinuation of MET TKI or EGFR TKI in the analysis.

**Table 3:** Adverse Event of Combination Treatments With EGFR TKI and MET TKI

TRAEs	Any Grade	Grades 1–2	Grades 3–4
Hypoalbuminemia	12 (44.4)	12 (44.4)	0
Creatinine increasing	12 (44.4)	12 (44.4)	0
Peripheral edema	12 (44.4)	11 (40.7)	1 (3.7)
Skin rash	8 (29.6)	8 (29.6)	0
Acne	7 (25.9)	7 (25.9)	0
Nausea	5 (18.5)	5 (18.5)	0
Constipation	5 (18.5)	5 (18.5)	0
Paronychia	4 (14.8)	4 (14.8)	0
Diarrhea	4 (14.8)	4 (14.8)	0
Stomatitis	4 (14.8)	4 (14.8)	0
Folliculitis	1 (3.7)	1 (3.7)	0
Thrombocytopenia	1 (3.7)	1 (3.7)	0

*Note:* All values are given in n (%).

TKI, tyrosine kinase inhibitor; TRAE, treatment-related adverse event.

### The Concurrent Alterations

**Figure 3:**
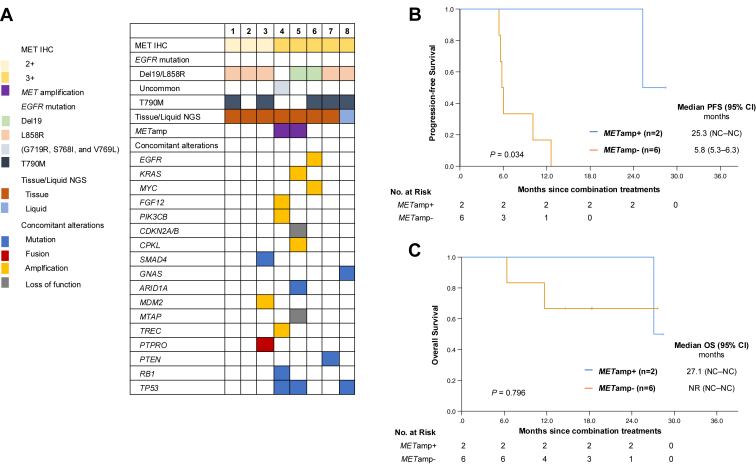
Results of next-generation sequencing *(A)*. Progression-free survival *(B)* and overall survival *(C)* comparing those with *MET*amp and without *MET*amp. Patients with *MET*amp reported better PFS than those without *MET*amp. CI, confidence interval; IHC, immunohistochemistry; NC, could not be calculated; NGS, next-generation sequencing; *MET*amp, *MET* amplification; NR, not reached; OS, overall survival; PFS, progression-free survival; TKI, tyrosine kinase inhibitor.

Among the 27 patients with MET overexpressed, eight underwent NGS examination after disease progression on EGFR TKI. The results of concurrent alterations are shown in Figure 3*A*–*C*, with seven patients having tissue NGS and one having liquid NGS. *MET*amp was detected in two patients (25.0%), who also had MET IHC 3 or higher. Meanwhile, *TP53* mutations were positive in three patients (37.5%). The characteristics and treatment outcomes of the combination therapy of the eight patients are reported in Supplementary Tables 2 and 3. The survival outcomes analysis reported that those with *MET*amp had a longer mPFS than those without *MET*amp (25.3 versus 5.8 mo; *p* = 0.034) (Fig. 3*B*), whereas there were no significant differences in the mOS (*MET*amp^+^: 27.1 mo versus *MET*amp^−^ : not reached mo; *p* = 0.796).

## Discussion

This study reported the efficacy and safety of combination therapy with EGFR TKIs and MET TKIs in patients with *EGFR*-mutant LUAD who were resistant to EGFR TKIs and positive for MET overexpression. This real-world evidence revealed an ORR of 29.6%, a DCR of 85.2%, an mPFS of 7.3 months, and an mOS of 26.9 months with combination therapy in 27 patients. In addition, 14 (51.9%) of these patients received chemotherapy previously, suggesting the activity of combination therapy in heavily treated patients.

In randomized controlled studies, combination therapy with EGFR TKI and MET TKI reported an ORR of 33% to 67%, a DCR of 50% to 90%, and an mPFS of five to 17 months.^16^, ^17^, ^18^^,^^20^, ^21^, ^22^ For those with *MET*amp, recent real-world studies reported outcomes of TKI combination therapy as an ORR of 35% to 48%, a DCR of 82% to 88%, and a PFS of 5.0 to 9.8 months.^23^, ^24^, ^25^ Another retrospective cohort, analyzing the treatment response of EGFR TKIs plus crizotinib in 11 patients with *MET*amp progression after EGFR TKI, reported a high ORR of 81.8%.^26^ In this study, the DCR and PFS were similar to previous studies, but the ORR was lower. Meanwhile, 44% of patients underwent surgical resection or radiotherapy for the primary tumor. Therefore, the residual tumor may be difficult to measure, and a high proportion of patients had stable disease after combination treatments, which may result in a lower ORR.

The treatment-related AEs of combination therapy have been reported in 88% to 100% of any grades and 34% to 57% of grade 3 or greater events.^17^^,^^20^^,^^22^ Diarrhea (48 to 58%) was more common when combined with tepotinib,^20^^,^^22^ whereas nausea (33%–41%) was more common when combined with capmabinib or salvolitinib.^17^^,^^21^ In the real-world setting, a previous study reported neutropenia (13.2%) and gastrointestinal upset (10.5%) as the most common treatment toxicities when combining EGFR TKI with crizotinib.^25^ Another study reported increased serum creatinine (39%), nausea (35%), and fatigue (21%) as the most common treatment toxicities.^24^ In this study, hypoalbuminemia (44.4%), peripheral edema (44.4%), and increased creatinine levels (44.4%) were the most common AEs. Only one patient experienced a grade 3 or greater event, which was peripheral edema. Symptoms such as diarrhea, nausea, or skin rash were well tolerated. In this study, all patients had previously undergone EGFR TKI treatment and may under-report symptoms they are accustomed to when taking EGFR TKI. Findings such as hypoalbuminemia and increased creatinine levels were detected through laboratory exams and were well documented. Peripheral edema is more easily detected during clinical visits. The events of dose reduction may be underestimated in a real-world setting, as patients might not report such behavior precisely.

In previous studies of the combination of EGFR TKI and MET TKI as a treatment option after the failure of first-line EGFR TKI, several biomarkers have been suggested as predictive factors of the treatment response. In the Tepotinib plus gefitinib in patients with EGFR-mutant non-small-cell lung cancer with MET overexpression or MET amplification and acquired resistance to previous EGFR inhibitor (INSIGHT study), both MET-IHC 3 or higher and positive *MET*amp (fluorescence in situ hybridization [FISH] gene copy number [GCN] ≥ 5) predicted the superiority of combination treatments (gefitinib plus tepotinib) over chemotherapy.^16^^,^^20^ In the SAVANNAH study, MET-IHC 90 or higher (3+ staining in ≥90% tumor cells) or MET FISH10+ (GCN ≥ 10) were associated with the survival benefits of combining osimertinib and savolitinib (ORR = 49%, mPFS = 7.1 mo).^27^ Meanwhile, tepotinib plus osimertinib has revealed promising activity in those with *MET*amp detected by FISH (GCN ≥ 5) or liquid NGS (plasma GNC ≥ 2.3) in the INSIGHT2 study.^22^ In addition, in the INSIGHT2 study, positive *TP53* mutations, detected in 49% of patients (21/43) with co-occurring alterations, reported no association with the treatment response.^22^ In real-world studies, positive *TP53* mutations were detected in 59% to 83% in patients with *EGFR*-mutant *MET*amp NSCLC.^23^^,^^25^ In our study, *TP53* mutations were the most common concomitant alterations, occurring in 37.5% of patients (3/8) studied using NGS. Meanwhile, a positive *MET*amp was associated with a longer PFS of combination therapy than those with negative *MET*amp (25.3 versus 5.8 mo; *p* = 0.034), which was compatible with previous studies.^18^^,^^20^^,^^22^

In addition to TKI combinations, novel agents, including bispecific antibodies and antibody-drug conjugates (ADCs), have also been studied in patients with *EGFR*-mutant NSCLC. In the phase 3 MARIPOSA-2 trial, the combination of amivantamab, a bispecific antibody targeting MET and EGFR receptors, plus chemotherapy significantly improved PFS compared with chemotherapy alone (6.3 versus 4.2 mo; Hazard ratio = 0.48; *p* < 0.001) in patients with disease progression on osimertinib.^28^ In addition to the combination of amivantamab plus chemotherapy, which reported a clinical benefit as second-line treatment in nonselective patients, another combination regimen of amivantamab with lazerinib also revealed MET-targeted activities. In cohort D of the CHRYSALIS-2 study, the combination therapy of amivantamab and lazertinib reported an ORR of 61% and mPFS of 12.2 months in patients with MET-positive (MET 3 or higher staining in ≥25% tumor cells) *EGFR*-mutant NSCLC progression on osimertinib.^29^ Teliso-V, a MET-targeted ADC, combined with osimertinib, reported an ORR of 50% and a mPFS of 6.8 months in patients with MET overexpressed (MET 3 or higher staining in ≥25% tumor cells) *EGFR*-mutant NSCLC progression on osimertinib.^30^ Although the combination of amivantamab and chemotherapy has become a new standard of care as second-line treatment for patients with advanced-stage *EGFR*-mutant NSCLC, this regimen also carries high rates of AEs, with 58% of patients reporting infusion-related reaction (all-grades), and 72% of patients had grade 3 or greater events.^28^ In addition, regimens needing intravenous infusion were more inconvenient than oral TKIs. As a result, the efficacy of the combination therapy of EGFR TKI with MET TKI was still worth investigating.

This study has several limitations. First, because this was a retrospective study and the data were from a single medical center, selection bias was inevitable. In this study, 88.9% of the patients had never smoked, which was higher than the 67% to 72% in previous studies.^16^^,^^22^ In Taiwan, more than half of lung cancer patients have never smoked.^31^ In addition, about 64% of patients with *EGFR* mutations have never smoked.^32^ As a result, the characteristics of patients in this study may differ from those in countries where lung cancer is primarily attributed to tobacco smoking, and the results may not be generalized to those areas. Second, in the real-world setting, the frequency of tumor assessment was not as regular as in clinical trials. As a result, strict adherence to RECIST may not be feasible, and most of the treatment responses were evaluated as stable disease. This may be another reason for the lower ORR in this study compared with previous studies. Third, the molecular analysis of concurrent alterations was only available for eight patients, so the exact concordance between MET overexpression and *MET*amp was not presented in this study. In addition, the detection of *MET*amp by NGS was not validated by FISH. In Taiwan, NGS has not been fully reimbursed for patients with lung cancer, nor have novel agents targeted acquired resistance to EGFR TKI. As a result, the molecular study of acquired EGFR TKI resistance was based on the availability of tissue, the performance status, and the financial status of the patients. These factors contributed to a low rate of NGS in this study. In a previous study, *MET*amp detected by FISH reported a superior predictive value for the clinical response.^33^ Nevertheless, the tools to detect *MET* alterations have been inconsistent across different studies. For MET overexpression, different detection assays have been used, such as D1C1 antibody in the INSIGHT study and SP44 in the TATTON study.^16^^,^^18^ Regarding *MET*amp, individuals with a *MET* GCN of 5 to 10 by FISH or NGS on tissue biopsy or a GCN of 2.3 to 2.5 in liquid biopsy have been enrolled in previous studies.^7^^,^^16^, ^17^, ^18^^,^^34^ In the ORCHARD study, multiple NGS platforms were used to detect *MET*amp, and the GCN detected ranged from 7 to 68.^34^ In the GEOMETRY mono-1 study, using FoundationOne CDx to detect *MET* alterations, a GCN of 6 or higher was regarded as positive for *MET*amp.^14^ For liquid biopsy, the cutoff values were GCN values of 2.5 or higher in the VISION study using Guardant360 CDx^15^ and 2.3 or higher in the INSIGHT 2 using the Archer platform.^35^^,^^36^ Thus, there is no universal diagnostic criteria when using different diagnostic platforms. Moreover, clinical waxing and waning of *MET* GCN in response to EGFR TKI selection pressure have been observed, and the exact GCN suggestive of *MET* dependence in the setting of *EGFR* acquired resistance may need to be clarified in future studies.^37^ Last but not least, this study enrolled a small sample size, and the follow-up duration may not be long enough. The OS events occurred in only 14 patients (51.9%) overall and three (37.5%) with an NGS test. As a result, although patients with MET-IHC 3 or higher reported a trend of longer OS than those with MET-IHC 2 or higher by Kaplan-Meier curve, the log-rank analysis revealed no significant difference. For patients with *MET*amp^+^, the significance of longer mPFS compared with those with *MET*amp^−^ did not translate to longer OS. This may also be attributed to the limitation of sample size and follow-up duration. In addition, as there were more upcoming novel agents for *EGFR*-mutant NSCLC, such as ADCs, immune checkpoint inhibitors, and amivantamab, the heterogenicity of subsequent treatment of these patients can also interfere the OS.

## Conclusion

For patients with *EGFR*-mutant MET-overexpressed LUAD experiencing resistance to EGFR TKIs, this study provided real-world data on combination therapy with EGFR TKIs and MET TKIs, demonstrating clinical activity and good tolerability.

## CRediT Authorship Contribution Statement

**Jia-Jun Wu**: Conceptualization, Software, Methodology, Formal analysis, Data curation, Writing - original draft, Visualization, Final approval.

**Zhe-Rong Zheng**: Methodology, Investigation, Data curation, Writing - original draft, Final approval.

**Tse-Hsien Lo**: Methodology, Investigation, Data curation, Final approval.

**Cheng-Hsiang Chu**: Formal analysis, Investigation, Data curation, Visualization, Writing - original draft, Final approval.

**Kun-Chieh Chen**: Conceptualization, Methodology, Resources, Writing - review & editing, Supervision, Project administration, Funding acquisition, Final approval.

**Gee-Chen Chang**: Conceptualization, Software, Methodology, Resources, Formal analysis, Data curation, Writing - original draft, Visualization, Writing - review & editing, Supervision, Project administration, Funding acquisition, Final approval.

## Ethical Approval

This study was approved by the Institutional Review Board of Chung Shan Medical University Hosptial.

## Informed Consent Statement

Written informed consent to access clinical data records was obtained from all patients.

## Data Availability Statement

The data generated in this study are available on reasonable request from the corresponding author.

## Disclosure

The authors declare no conflict of interest.
