# Supplementary material for: Combination Therapy With MET Tyrosine Kinase Inhibitor and EGFR Tyrosine Kinase Inhibitor in Patients With MET-Overexpressed EGFR-Mutant Lung Adenocarcinoma
Source: JTO Clin Res Rep. 2025 Apr 9;6(6):100832. doi: 10.1016/j.jtocrr.2025.100832 (PMC12133690; doi:10.1016/j.jtocrr.2025.100832)
Supplement: Supplementary Table 1-3 and Supplementary Figure 1 [file mmc1.pdf]

Table S1. Univariate and multivariate analysis of factors on survival of MET TKI

PFS	Univariate		Multivariate	
Factors	HR (95% CI)	*P* value[a]	HR (95% CI)	*P* value[a]
Age				
≥65 vs ＜65 years	0.62 (0.26–1.44)	0.262	0.58 (0.23–1.52)	0.269
Sex				
Male vs Female	1.41 (0.61–3.29)	0.425	1.63 (0.54–4.96)	0.390
Smoking				
Smoked vs Never-smoked	0.87 (0.26–2.99)	0.829		
ECOG PS				
0–1 vs ≥2	1.26 (0.29–5.43)	0.753		
Stage				
IVA vs IVB	0.93 (0.40–2.18)	0.865	0.73 (0.26–2.06)	0.549
Brain metastasis				
Yes vs No	0.90 (0.26–3.08)	0.862		
Prior Chemotherapy				
Yes vs No	0.83 (0.36–1.94)	0.666	0.67 (0.23–1.95)	0.457
Primary tumor consolidation therapy				
Yes vs No	0.45 (0.18–1.12)	0.086	0.37 (0.13–1.06)	0.064
T790M				
positive vs negative	2.18 (0.93–5.13)	0.074	2.26 (0.81–6.34)	0.120
MET-IHC				
3+ vs 2+	0.73 (0.31 – 1.71)	0.473	1.11 (0.42–2.90)	0.838
**OS**	**Univariate**		**Multivariate**	
Factors	HR (95% CI)	*P* value[a]	HR (95% CI)	*P* value[a]
Age				
≥65 vs ＜65 years	0.85 (0.28–2.53)	0.763	0.74 (0.21–2.64)	0.646
Sex				
Male vs Female	1.30 (0.45–3.73)	0.624	1.88 (0.45–7.93)	0.391
Smoking				
Smoked vs Never-smoked	1.05 (0.23–4.72)	0.953		
ECOG PS				
0–1 vs ≥2	0.48 (0.11–2.18)	0.342		
Stage				
IVA vs IVB	0.68 (0.23–2.03)	0.483	0.76 (0.23–2.58)	0.663
Brain metastasis				
Yes vs No	0.43 (0.06–3.29)	0.414		
Prior Chemotherapy				

Yes vs No	1.04 (0.37–2.98)	0.939	0.87 (0.23–3.30)	0.837
Primary tumor consolidation therapy				
Yes vs No	0.52 (0.17–1.61)	0.254	0.42 (0.11–1.56)	0.194
T790M				
positive vs negative	0.58 (0.18–1.85)	0.355	0.40 (0.10–1.53)	0.179
MET-IHC				
3+ vs 2+	0.65 (0.22–1.91)	0.435	0.67 (0.18–2.51)	0.555

[a]*P* value: By Cox proportional-hazards model.

CI, confidence interval; ECOG PS, Eastern Cooperative Oncology Group performance status; HR, hazard ratio; *MET*, mesenchymal–epithelial transition factor; OS, overall survival; PFS, progression-free survival; TKI, tyrosine kinase inhibitor.

Table S2. Characteristics for those with NGS data

Variables	N=8
Age, median (IQR)	63.0 (50.5 – 70.0)
Sex	
Male	1 (12.5%)
Female	7 (87.5%)
Smoking	
Never smoked	7 (87.5%)
Smoked	1 (12.5%)
ECOG PS	
0–1	7 (87.5%)
≥2	1 (12.5%)
Stage	
IVA	5 (62.5%)
IVB	3 (37.5%)
NGS method	
Tissue	7 (12.5%)
Liquid	1 (87.5%)
*MET*amp	2 (25.0%)
TP53 mutation	3 (37.5%)

Categorical data are presented as number (%).

ECOG PS, Eastern Cooperative Oncology Group performance status; *EGFR*, Epidermal growth factor receptor; IHC, immunohistochemistry; *MET*, mesenchymal–epithelial transition factor; IQR, interquartile range; TKI, tyrosine kinase inhibitor.

Table S3. Treatment outcomes for those with NGS data

Outcomes	All<br>N=8	*MET*amp^{+}<br>N=2	*MET*amp^{-}<br>N=6
Survival time, median (95% CI)			
PFS, months	6.0 (0.1–12.0)	25.3 (NC–NC)	5.8 (5.3–6.3)
OS, months	27.1 (NC–NC)	27.1 (NC–NC)	NR (NC–NC)
Treatment response, number (%)			
PR	1 (12.5%)	0	1 (16.7%)
SD	7 (87.5%)	2 (100%)	5 (83.3%)
PD	0	0	0
ORR	12.5%	0%	16.7%
DCR	100%	100%	100%

CI, confidence interval; DCR, disease control rate; *MET*, mesenchymal–epithelial transition factor; NC, could not be calculated; NR, not reached; ORR, objective response rate; OS, overall survival; PFS, progression-free survival; TKI, tyrosine kinase inhibitor.

Figure S1. Examples for MET-IHC

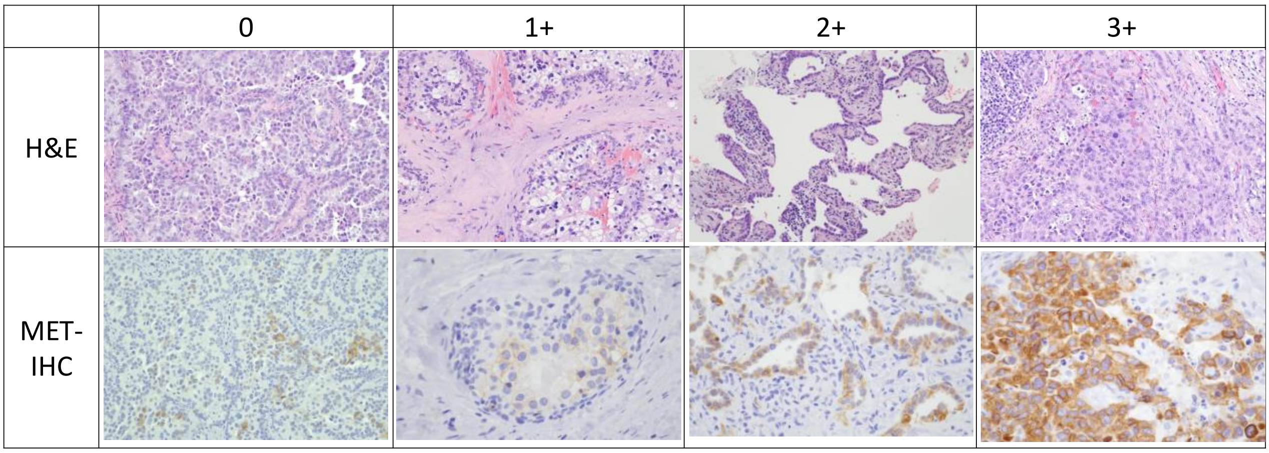

H&E, Hematoxylin and eosin staining; IHC, immunohistochemistry staining; MET, Mesenchymal-epithelial transition factor.
